# Triploid loquat maintains photosynthetic stability under freezing stress through excessive accumulation of unsaturated lipids

**DOI:** 10.1093/hr/uhag096

**Published:** 2026-03-16

**Authors:** Mingxiu Liu, Xiaodong Suo, Xun Xu, Hao Yang, Mubasshir Hussain, Danlong Jing, Jiangbo Dang, Di Wu, Shuming Wang, Yan Xia, Qiao He, Guolu Liang, Qigao Guo

**Affiliations:** Key Laboratory of Agricultural Biosafety and Green Production of Upper Yangtze River (Ministry of Education), Southwest University, Chongqing 400715, China; Chongqing Key Laboratory of Forest Ecological Restoration and Utilization in the Three Gorges Reservoir Area, Southwest University, Chongqing 400715, China; College of Horticulture and Landscape Architecture, Southwest University, Beibei, Chongqing 400715, China; College of Animal Science and Technology, Southwest University, Chongqing 400716, China; Key Laboratory of Agricultural Biosafety and Green Production of Upper Yangtze River (Ministry of Education), Southwest University, Chongqing 400715, China; Chongqing Key Laboratory of Forest Ecological Restoration and Utilization in the Three Gorges Reservoir Area, Southwest University, Chongqing 400715, China; College of Horticulture and Landscape Architecture, Southwest University, Beibei, Chongqing 400715, China; Key Laboratory of Agricultural Biosafety and Green Production of Upper Yangtze River (Ministry of Education), Southwest University, Chongqing 400715, China; Chongqing Key Laboratory of Forest Ecological Restoration and Utilization in the Three Gorges Reservoir Area, Southwest University, Chongqing 400715, China; College of Horticulture and Landscape Architecture, Southwest University, Beibei, Chongqing 400715, China; College of Plant Science & Technology, Huazhong Agricultural University, Wuhan 430070, China; Key Laboratory of Agricultural Biosafety and Green Production of Upper Yangtze River (Ministry of Education), Southwest University, Chongqing 400715, China; Chongqing Key Laboratory of Forest Ecological Restoration and Utilization in the Three Gorges Reservoir Area, Southwest University, Chongqing 400715, China; College of Horticulture and Landscape Architecture, Southwest University, Beibei, Chongqing 400715, China; Key Laboratory of Agricultural Biosafety and Green Production of Upper Yangtze River (Ministry of Education), Southwest University, Chongqing 400715, China; Chongqing Key Laboratory of Forest Ecological Restoration and Utilization in the Three Gorges Reservoir Area, Southwest University, Chongqing 400715, China; College of Horticulture and Landscape Architecture, Southwest University, Beibei, Chongqing 400715, China; Key Laboratory of Agricultural Biosafety and Green Production of Upper Yangtze River (Ministry of Education), Southwest University, Chongqing 400715, China; Chongqing Key Laboratory of Forest Ecological Restoration and Utilization in the Three Gorges Reservoir Area, Southwest University, Chongqing 400715, China; College of Horticulture and Landscape Architecture, Southwest University, Beibei, Chongqing 400715, China; Key Laboratory of Agricultural Biosafety and Green Production of Upper Yangtze River (Ministry of Education), Southwest University, Chongqing 400715, China; Chongqing Key Laboratory of Forest Ecological Restoration and Utilization in the Three Gorges Reservoir Area, Southwest University, Chongqing 400715, China; College of Horticulture and Landscape Architecture, Southwest University, Beibei, Chongqing 400715, China; Key Laboratory of Agricultural Biosafety and Green Production of Upper Yangtze River (Ministry of Education), Southwest University, Chongqing 400715, China; Chongqing Key Laboratory of Forest Ecological Restoration and Utilization in the Three Gorges Reservoir Area, Southwest University, Chongqing 400715, China; College of Horticulture and Landscape Architecture, Southwest University, Beibei, Chongqing 400715, China; Key Laboratory of Agricultural Biosafety and Green Production of Upper Yangtze River (Ministry of Education), Southwest University, Chongqing 400715, China; Chongqing Key Laboratory of Forest Ecological Restoration and Utilization in the Three Gorges Reservoir Area, Southwest University, Chongqing 400715, China; College of Horticulture and Landscape Architecture, Southwest University, Beibei, Chongqing 400715, China; Key Laboratory of Agricultural Biosafety and Green Production of Upper Yangtze River (Ministry of Education), Southwest University, Chongqing 400715, China; Chongqing Key Laboratory of Forest Ecological Restoration and Utilization in the Three Gorges Reservoir Area, Southwest University, Chongqing 400715, China; College of Horticulture and Landscape Architecture, Southwest University, Beibei, Chongqing 400715, China; Key Laboratory of Agricultural Biosafety and Green Production of Upper Yangtze River (Ministry of Education), Southwest University, Chongqing 400715, China; Chongqing Key Laboratory of Forest Ecological Restoration and Utilization in the Three Gorges Reservoir Area, Southwest University, Chongqing 400715, China; College of Horticulture and Landscape Architecture, Southwest University, Beibei, Chongqing 400715, China; Key Laboratory of Agricultural Biosafety and Green Production of Upper Yangtze River (Ministry of Education), Southwest University, Chongqing 400715, China; Chongqing Key Laboratory of Forest Ecological Restoration and Utilization in the Three Gorges Reservoir Area, Southwest University, Chongqing 400715, China; College of Horticulture and Landscape Architecture, Southwest University, Beibei, Chongqing 400715, China; Key Laboratory of Agricultural Biosafety and Green Production of Upper Yangtze River (Ministry of Education), Southwest University, Chongqing 400715, China; Chongqing Key Laboratory of Forest Ecological Restoration and Utilization in the Three Gorges Reservoir Area, Southwest University, Chongqing 400715, China; College of Horticulture and Landscape Architecture, Southwest University, Beibei, Chongqing 400715, China

## Abstract

Loquat (*Eriobotrya japonica* Lindl.), a subtropical evergreen species of the Rosaceae family, faces industry constraints in industrial development due to its sensitivity to freezing temperatures and low photosynthetic efficiency. Polyploid loquats, particularly triploids, exhibit enhanced stress resistance, vigorous growth, and seedless fruit production. In this study, triploid F1 progeny (B431 × GZ23) was obtained through hybridization between diploid (GZ23) and tetraploid (B431) parents. Under −3°C stress, the triploid lines exhibited significantly improved freezing tolerance and photosynthetic performance, as evidenced by chlorophyll fluorescence parameters and ultrastructural integrity. Lipidomics profiling across all B431 × GZ23 lines revealed that phosphatidylcholine (PC), particularly the abundant unsaturated PC species 18:2/18:2, played a key role in these adaptive advantages. Compared to the parental lines, *EjFAD2* expression was specifically upregulated in triploid loquats under freezing stress. Consistent lipidomic and gene expression patterns across three B431 × GZ23 lines ruled out line-specific mutations. Heterologous expression of *EjFAD2* in *Arabidopsis* increased freezing tolerance. Co-expression analysis identified *EjMYBS3* as a regulator that binds to the *EjFAD2* promoter, and its overexpression in transgenic *Arabidopsis* enhanced freezing tolerance. Transient expression of *EjFAD2* and *EjMYBS3* increased the content of PC 18:2/18:2 in loquat, which contributed to the maintenance of photosystem activity under freezing stress, thereby enhancing the freezing tolerance of loquat. Collectively, these findings provide preliminary insights into the molecular mechanisms underlying cold resistance in polyploid loquats and highlight the regulatory role of *EjFAD2* and *EjMYBS3* in freezing stress response.

## Introduction

Frequent extreme climatic events subject plants to severe biotic stresses (e.g. from fungi and insects) and abiotic stresses (such as cold, drought, and salinity), consequently impairing their growth [1, 2]. As a critical regulator of plant development, temperature significantly influences growth. Among temperature stresses, low-temperature stress—particularly freezing conditions—limits species distribution and agricultural productivity by disrupting membrane fluidity, water and nutrient uptake, and other physiological processes [3]. Therefore, elucidating cold tolerance mechanisms and developing cold-resistant germplasm can facilitate the expansion of cultivation areas and improve crop yields [4, 5]. Given the agricultural significance of freezing stress, research in this area has become a major focus in plant science.

Polyploidy serves as an adaptive evolutionary strategy that mitigates abiotic stress [6, 7]. Since Lutz’s discovery of the tetraploid *gigas* mutant in 1907, polyploidy has been established as a key driver of plant evolutionary diversification [8]. In agriculture contexts, gene duplications resulting from polyploidization serve as critical determinants of stress resilience [9]. Polyploidy influences various phenotypic traits, such as increasing organ biomass (leaves, fruits) and enhancing tolerance to a range of abiotic stresses, including extreme temperatures, drought, and fluctuating light conditions (either excessive or insufficient) [10]. Consequently, polyploid plants generally exhibit superior cold resistance and higher yields compared to their diploid counterparts [11]. The association between abiotic stress and polyploid has been studied for a long time, particularly in cold environments. However, only a limited number of studies have specifically investigated the relationship between polyploidy and freezing stress [12].

Research on plant freezing stress responses has advanced considerably. Chilling tolerance involves cellular structural remodeling and regulation of gene expression [13], while freezing stress primarily compromises membrane integrity and function. Numerous studies associate chilling injury with membrane lipid fluidity [14, 15], where the levels of unsaturated fatty acid play a key role in modulating fluidity. Increased unsaturation is a widely observed adaptation to low temperatures [16, 17], and high thylakoid lipid unsaturation has been shown to enhance recovery from photoinhibition [18, 19]. Membrane desaturation is catalyzed by fatty acid desaturases (FADs): FAD2 and FAD3 mediate extraplastidic desaturation, producing C18:2/C18:3 [20], whereas FAD4 to FAD8 regulate desaturation in the chloroplast inner membrane [21]. The expression of *FAD2*, *FAD3*, *FAD6*, and *FAD8* is induced by low temperatures across various plant species [22–24], and *fad5/fad6* mutants display defective chloroplast development under cold stress [25]. Zhou *et al.* reported that overexpression of *FAD2*, *FAD3*, and *FAD8* enhances freezing tolerance in plants [26].

Loquat (*Eriobotrya japonica* Lindl.) is a perennial evergreen species belonging to the Rosaceae family [27]. Due to its relatively low photosynthetic activity under cold conditions, loquat yield is often severely reduced under freezing stress. Therefore, developing loquat varieties with enhanced abiotic stress resistance, high edible quality, and efficient photosynthetic capacity is crucial for addressing challenges facing the loquat industry. Triploid loquats have been shown to exhibit significantly enhanced stress resistance and adaptability compared to diploid loquats [28]. Thus, it is essential to investigate the freezing stress tolerance of triploid loquats to support the sustainable development of the industry.

Our prior work generated triploids through diploid–tetraploid hybridization, which revealed that superior photosystem functionality contributes to their high photosynthetic efficiency [28]. In this study, we found that the triploid exhibits the strongest frost resistance and is capable of maintaining photosynthetic activity at −3°C. Lipidomic analysis confirmed that increased membrane lipid unsaturation is crucial for sustaining photosynthesis under freezing stress in these triploids. Transcriptome analysis further indicated that *EjFAD2* is a key factor for maintaining photosynthesis under freezing stress conditions. Heterologous expression of *EjFAD2* in *Arabidopsis* increased lipid unsaturation and thereby supported photosynthesis under freezing stress. Concurrently, co-expression analysis identified EjMYBS3 as an interacting partner of EjFAD2, and its heterologous expression in *Arabidopsis* enhanced freezing tolerance in transgenic plants. Altogether, our findings provide novel insights into the regulatory mechanisms underlying the maintenance of photosynthesis in triploids under freezing stress.

## Results

### Triploid loquat exhibited stable photosynthetic efficiency under freezing stress

**Figure 1:**
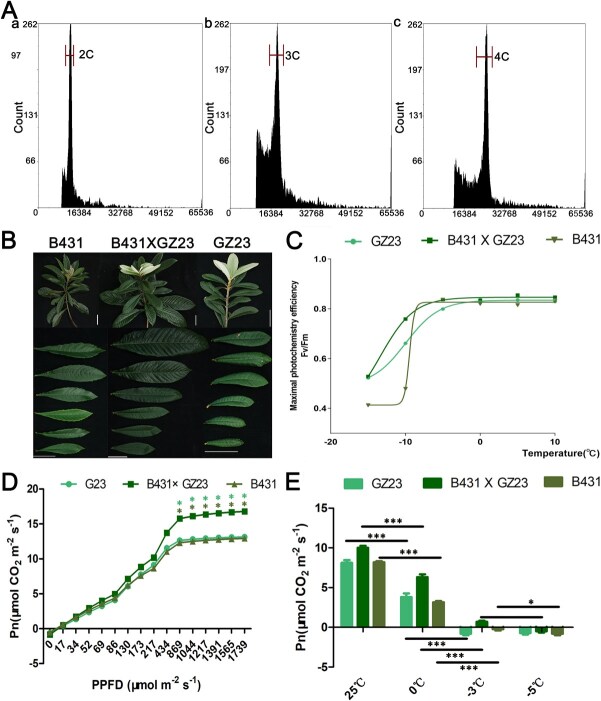
Analysis of freezing resistance and photosynthetic efficiency in triploid loquat and its parental lines. (A) Flow cytometric histograms of DAPI-stained leaf nuclei from loquat of different ploidy levels: a, diploid, GZ23; b, triploid, B431 × GZ23; c, tetraploid, B431. (B) Phenotypic characteristics of triploid loquat and its parents. Scale bar = 10 cm. (C) S-curves fitted to Fv/Fm of triploid loquat and its parents under different temperature. (D) S-curves fitted to *Fv/Fm* of triploid loquat and its parents under different temperature Light response curves of triploid loquat and its parents. (E) Variation in net photosynthetic rate of triploid loquat and its parents under different temperature conditions. Error bars represent the SD of three separate biological replicates. Data were analyzed using Student’s *t* test, with ^*^, ^**^, and ^***^ denoting significance at *P* < 0.05, *P* < 0.01, and *P* < 0.001, respectively.

To confirm the genotypes of different ploidy loquat materials, nuclei were isolated from leaves and analyzed by using a ploidy analyser (Fig. 1A and Fig. S1). Diploid samples exhibited a single peak at a fluorescence value corresponding to 2C DNA content. Triploid samples displayed a peak with a fluorescence value 1.5 times higher than diploids, while tetraploid samples showed a peak with twice the fluorescence value of diploids (Fig. 1A). Observations revealed that triploid leaves were significantly larger than those of diploid and tetraploid materials (Fig. 1B).

Photosynthetic efficiency analysis revealed that triploids exhibited a significantly higher *net photosynthetic rate* (*Pn*) across all light intensities compared to diploids and tetraploids, with tetraploids showing similar performance to diploids (Fig. 1D). Considering the established role of *Fv/Fm* as an indicator of stress, chlorophyll fluorescence combined with logistic modeling was employed to determine semilethal temperature (LT50). All ploidy lines maintained stable *Fv*/*Fm* values (~0.8) above −5°C (Fig. 1C and Table S1). However, *Fv*/*Fm* declined markedly below −10°C, reaching its lowest values at −15°C. Triploids showed the lowest LT50 (−13.35°C). Furthermore, measurements of *Pn* in loquat lines at varying temperatures indicated a progressive decline in *Pn* with decreasing temperature (Fig. 1E). When the temperature dropped to −3°C, all lines showed significant reductions compared to 0°C, although only the triploid maintained a positive *Pn* value. Upon further cooling to −5°C, *Pn* values for all lines became negative.

These results indicate that the triploid loquat exhibits characteristics of high growth potential, high photosynthetic efficiency, and strong freezing resistance.

### Triploid loquat maintains membrane stability to enhance its growth sustainability under freezing stress

**Figure 2:**
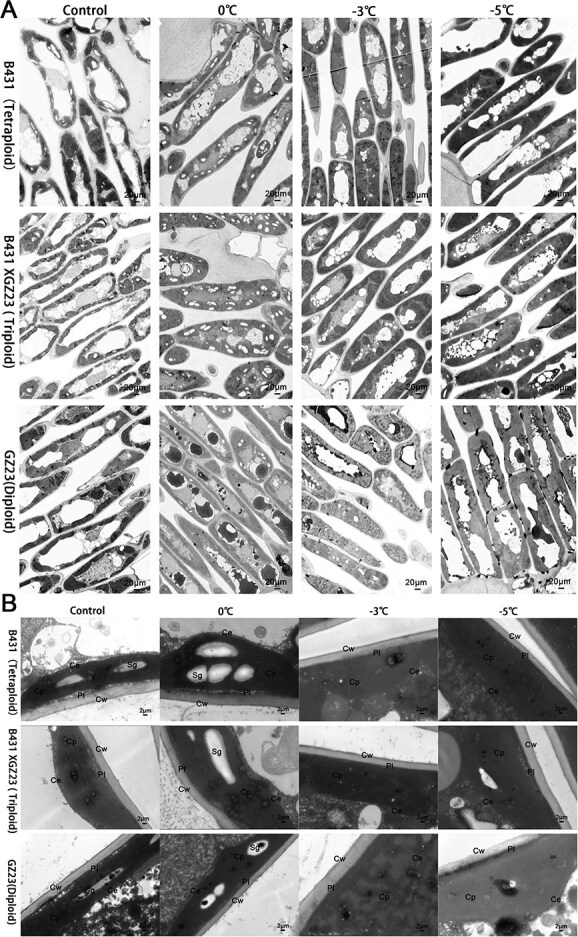
Cell ultrastructure of triploid loquat and its parents under freezing stress. (A) Leaf morphology and cell ultrastructure of different ploidy loquat materials under freezing stress. (B) Detailed local ultrastructure diagram. Diploid, GZ23-1; triploid, B431 × GZ23-1; tetraploid, B431-1. Cw, cell wall; Cp, chloroplast; Ce, chloroplast membrane; Pl, plasmalemma; Sg, starch granule.

To elucidate the relationships between photosynthesis and freezing tolerance across ploidy levels, transmission electron microscopy (TEM) was used to analyze the ultrastructure (Fig. 2). At 0°C, triploids exhibited a significantly higher number of chloroplast starch granules compared to diploids and tetraploids (Fig. S2A). At −3°C, triploids maintained intact chloroplast membranes with clearly defined boundaries, whereas tetraploids showed intact membranes with blurred boundaries, and diploids displayed extensive chloroplast membrane fusion. At −5°C, plasma membranes remained relatively intact across all genotypes; however, diploids displayed complete chloroplast dissociation, organelle degradation, and chloroplast–plasma membrane fusion, despite the preservation of overall cellular integrity. Statistical analyses of both chloroplast integrity and length-to-width ratio revealed significant differences consistent with the aforementioned results (Fig. S2B and C).

**Figure 3:**
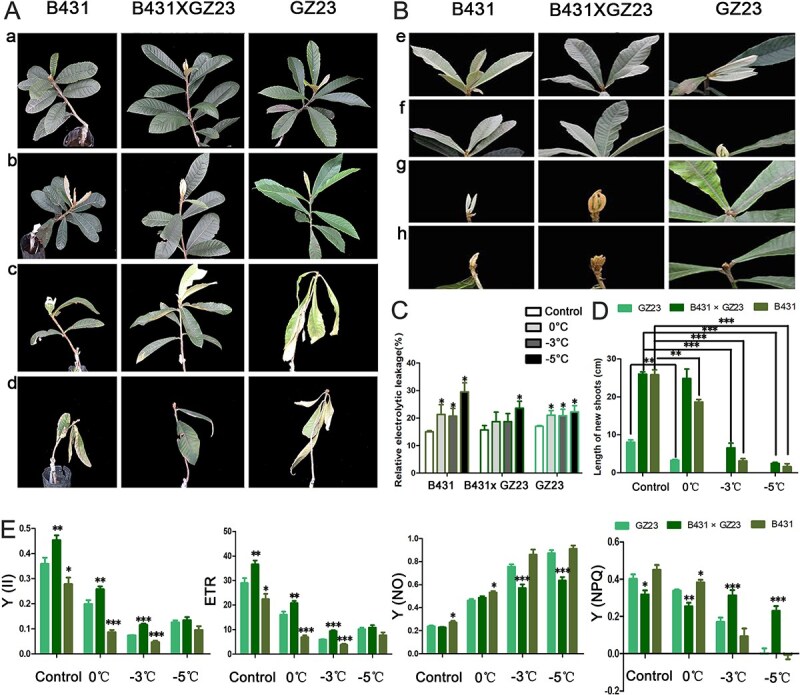
Growth status of triploid loquat and its parents after recovery at normal temperature for 60 days. (A) Plant growth status. a, Control at 25°C; b, 7 days after recovery from 0°C to 25°C; c, 60 days after recovery from −3°C to 25°C; d, 60 days after recovery from −5°C to 25°C. (B) Shoot growth status of triploid loquat and its parents after recovery at 25°C. e, Control at 25°C; f, 30 days after recovery from 0°C to 25°C; g, 30 days after recovery from −3°C to 25°C; h, 30 days after recovery from −5°C to 25°C. (C) Relative plasma membrane permeability under 72 h of freezing stress. (D) New shoot’s length of triploid loquat and its parents after recovery at 25°C for 30 days. (E) Analysis of chlorophyll fluorescence parameters in polyploid loquat at different temperatures. Genotypes: diploid (GZ23), triploid (B431 × GZ23), and tetraploid (B431). The SD of three separate biological replicates is shown by error bars. Data were analyzed using Student’s *t* test, with ^*^, ^**^, and ^***^ denoting significance at *P* < 0.05, *P* < 0.01, and *P* < 0.001, respectively.

After 72 h of treatment, the materials were transferred to 25°C. All ploidy levels resumed normal growth after 7 days at 0°C, and new shoot emergence after 30 days (Fig. 3A and B). When the temperature was increased from −3°C to 25°C, triploid loquat gradually returned to normal growth after 60 days with new shoots sprouting after 30 days. Tetraploid loquat also resumed normal growth after 30 days, although dehydrated patches appeared and a few leaves were shed. In contrast to the triploid and tetraploid plants, there were no new shoots from diploid loquat; meanwhile, the leaves gradually dried up and fell off after returning to 25°C, and all diploid plants died within 60 days. Exposure to −5°C severely affected all genotypes, resulting in complete defoliation. After 30 days, triploid and tetraploid loquat exhibited survival rates of 50% and 25%, respectively, while all diploid loquat plants died. The degree of damage to the cytoplasmic membrane was assessed by analyzing the relative permeability of plasma membrane following 72 h of freezing stress. Higher value indicated greater plasma membrane damage, both tetraploid and diploid loquat showed significant increases compared to controls after various freezing stress treatments, while triploid loquat exhibited a significant increase only after exposure to −5°C (Fig. 3C). Fluorescence parameters [including *Y*(II), *ETR*, and *Y*(NO)] were measured to assess the extent of damage to PS II, while *Y*(NPQ) was measured to evaluate the level of photosystem protection. As shown in Fig. 3E, these parameters exhibited significant alterations in triploids under freezing temperatures, indicating maintained stability of photosystem II activity during cold stress.

These results indicated that triploid loquat exhibited greater membrane stability under freezing stress compared to its parents, particularly in terms of chloroplast integrity, thereby maintaining cellular structure and function more effectively under freezing stress conditions.

### The content and degree of unsaturation of phosphatidylglycerol in triploid loquat lines (B431 **×** GZ23) increased significantly under freezing stress

**Figure 4:**
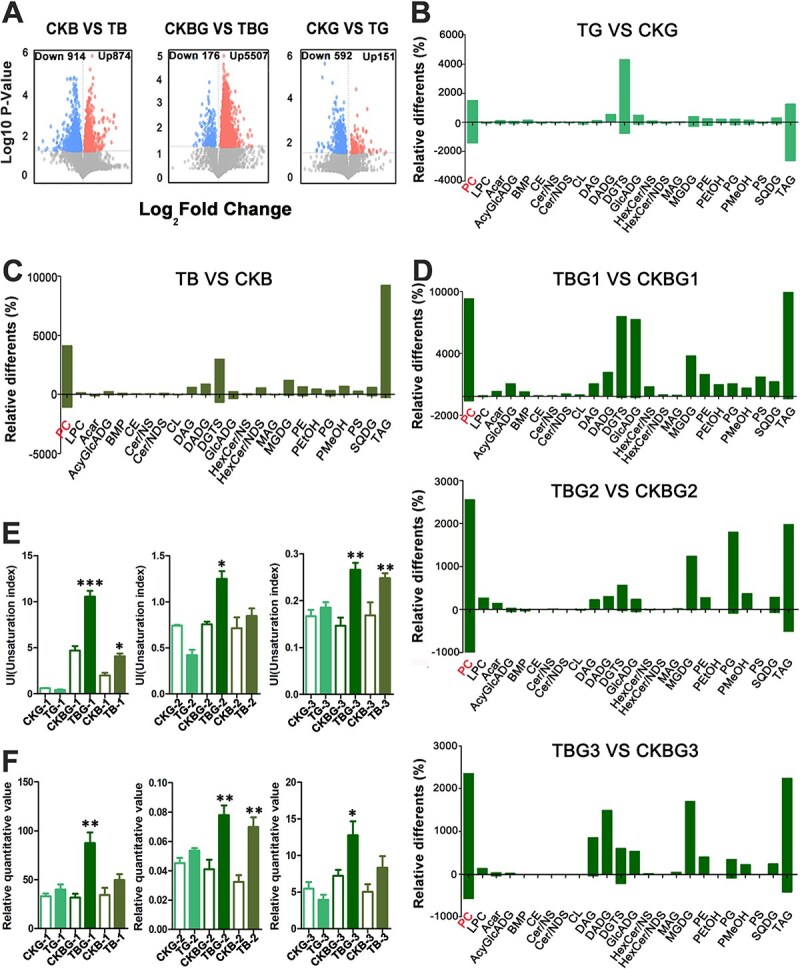
Changes in the lipid composition of each ploidy loquat under freezing stress. (A) The number of significantly altered lipids species across ploidy levels loquat under freezing stress. (B) Changes in the lipid composition of diploid loquat under freezing stress. (C) Changes in the lipid composition of tetraploid loquat under freezing stress. (D) Changes in the lipid composition of triploid loquat under freezing stress. (E) The unsaturation index (UI) of PC in each ploidy loquat under freezing stress. (F) Relative quantitative analysis of unsaturated molecules of PC (18:2/18:2) in each ploidy loquat under freezing stress. CK represents the control temperature 25°C; T represents −3°C. The SD of three separate biological replicates is shown by error bars. Data were analyzed using Student’s *t* test: ^*^, ^**^, and ^***^ indicate *P* < 0.05, *P* < 0.01, and *P* < 0.001, respectively.

The chloroplast membrane of triploid loquat exhibited a more intact and distinct structure compared to that of diploid and tetraploid under freezing stress (−3°C). To investigate the changes in lipid composition and content among diploid, tetraploid, and triploid loquats, we employed LC–MS/MS to quantify lipid levels in leaves from both control (25°C) and freezing-stressed conditions (−3°C). The results showed that 5683 lipids were significantly altered in triploid loquats after freezing stress, compared to 743 in diploid and 1788 in tetraploid (Fig. 4A). Notably, seven chloroplast membrane-associated lipid classes, including PC, PE, PG, TAG, DGDG, SQDG, and MGDG, showed significant variations. Among these, PC was one of the most significantly markedly altered lipids specifically in triploid loquats, differing from the patterns observed in diploid and tetraploid.

To eliminate the influence of individual genetic variation, lipid profiles were also analyzed in additional lines (B431 × GZ23-2 and B431 × GZ23-3), and the results were consistent with those obtained from B431 × GZ23-1 (Fig. 4B–D). The Unsaturation index (UI) of PC reached a peak value of 10.55 across all triploid lines (Fig. 4E). Relative quantitative analysis of PC 18:2/18:2 demonstrated an increase in all ploidy levels after freezing stress, with the most pronounced increase observed in triploid loquats (Fig. 4F).

These results suggest that phosphatidylcholine (PC), particularly PC 18:2/18:2, plays a key role in the loquat’s response to freezing stress, and that unsaturated PC contributes significantly to the freezing stress tolerance of triploid loquat.

### *EjFAD2* was involved in enhancing the unsaturation of PC in triploid loquat under freezing stress

**Figure 5:**
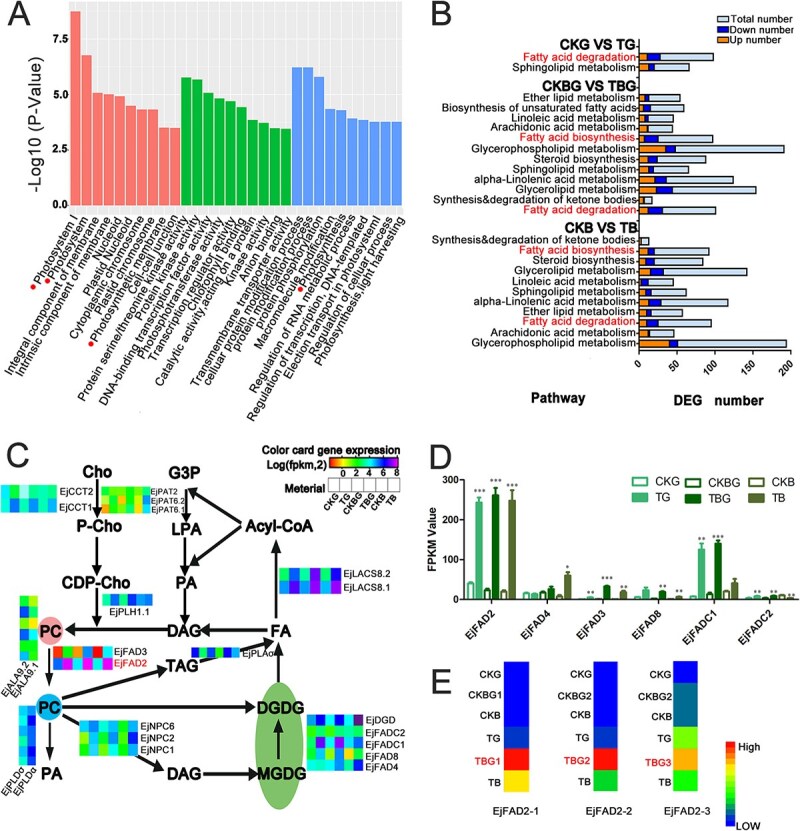
Lipid metabolism pathways of DEGs in triploid loquat and its parents under −3°C stress for 72 h. (A) GO classification of DEG in triploid loquat. (B) Comparative analysis of lipid metabolism pathways in triploid loquat and its parents under −3°C stress for 72 h. (C) Expression profiles of DEGs involved in lipid metabolic pathways. (D) Transcriptomic analysis of *FAD* gene expression in loquat materials. (E) Relative expression levels of *EjFAD2* in B431 × GZ23 progeny determined by RT-PCR. The SD of three separate biological replicates is shown by error bars. Data underwent Student’s *t* test: ^*^, ^**^, and ^***^ indicate *P* < 0.05, 0.001 < *P* < 0.01, and *P* < 0.001, respectively.

To further characterize the differentially expressed genes (DEGs) in triploid loquat and its parents under freezing stress, transcriptomic analysis was conducted. The results indicated that photosynthesis-related genes were the most significantly enriched in loquats of different ploidy levels, followed by genes associated with bio-membrane. These findings suggested that freezing stress had a greater impact on the photosynthesis and membrane systems of triploid loquats (Fig. 5A). To further explore lipid metabolism under freezing stress, DEGs related to lipid metabolism were analyzed. The numbers of enriched pathways in diploid, triploid, and tetraploid loquats were 2, 12, and 11, respectively. These results indicate that lipid metabolism in triploid and tetraploid loquats was more responsive to freezing stress, which is consistent with the lipid metabolome analysis results (Fig. 5B).

**Figure 6:**
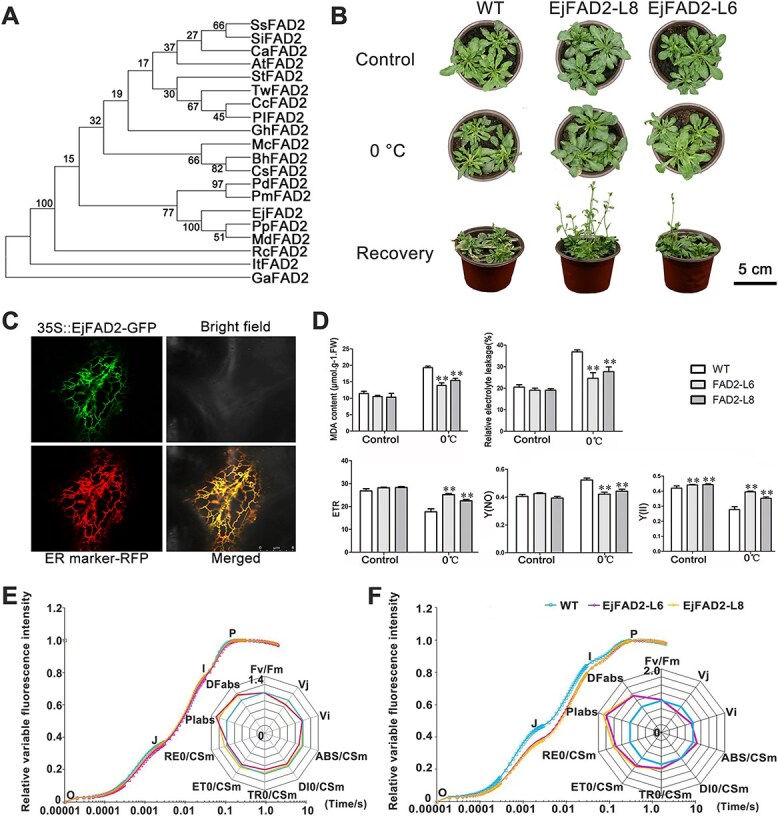
Heterologous expression of *EjFAD2* enhances the photosynthetic performance of *Arabidopsis* under freezing stress. (A) Phylogenetic analysis of EjFAD2 and related FAD2 proteins from other plant species. (B) Characterization of the response of *EjFAD2-*overexpressing *Arabidopsis* plants under freezing stress. (C) Subcellular localization of EjFAD2 in epidermal cells of *Nicotiana benthamiana*. (D) Fluorescence parameters of wild-type and *EjFAD2*-overexpressing *Arabidopsis* under freezing stress. (E) OJIP curves and fluorescence radar images of wild-type and *EjFAD2*-overexpressing *Arabidopsis* at 25°C. (F) OJIP curves and fluorescence radar images of wild-type and *EjFAD2-*overexpressing *Arabidopsis* at 0°C. Error bars represent the SD of three separate biological replicates. Data underwent Student’s *t* test: ^**^ indicates *P* < 0.01.

Considering the importance of PC, the DEGs associated with PC were analyzed. As shown in Fig. 6, six *FAD* genes were up-regulated after treatment at −3°C. Among the five *FAD* genes (*EjFAD2*, *EjFAD3*, *EjFAD8*, *EjFADC1*, and *EjFADC2*), the expression levels in triploid and tetraploid plants were higher than those in diploid plants under the control condition, and the up-regulation was most significant in triploid. These results were confirmed by qPCR (Fig. 5C and D). Analysis of gene expression in three lines of B431 **×** GZ23 revealed that *FAD2* exhibited the highest expression level under freezing stress (Fig. 5E).

These findings demonstrate that *FADs* are involved in the response to freezing stress, and *EjFAD2* plays a crucial role in the freezing tolerance of triploid loquat.

### Heterologous expression of *EjFAD2* enhances freezing tolerance in *Arabidopsis*

Sequence analysis of EjFAD2 revealed that it is highly homologous to MdFAD2 and PpFAD2 (Fig. 6A). Subcellular localization analysis indicated that EjFAD2 is an endoplasmic reticulum (ER)-localized protein. We overexpressed *EjFAD2* in *Arabidopsis thaliana* to characterize its functional role (Fig. 6B) (Fig. S4A). To investigate the biological function of EjFAD2, transgenic and wild-type plants were subjected to 0°C treatment followed by a 6-day recovery period under normal growth conditions. As shown in Fig. 6C, all plant lines exhibited normal growth under both normal temperature and cold treatment. However, after returning to normal temperature for 6 days, wild-type leaves displayed signs of wilting, whereas the transgenic lines remained healthy and continued to grow normally. These results demonstrate that heterologous expression of *EjFAD2* enhances frost tolerance in *Arabidopsis*.

To clarify the relationship between the photosynthetic system and plasma membrane stability under freezing stress, physiological analyses were conducted. First, electrolyte leakage and lipid peroxidation levels were measured to assess the extent of membrane damage. After 96 h at 0°C, transgenic lines exhibited significantly lower levels of electrolyte leakage and malondialdehyde (MDA) content compared to the wild-type (WT) control. Furthermore, fluorescence parameters were measured to evaluate the degree of damage to the photosynthetic apparatus. The results indicated that the *Y*(II), *ETR*, and *Y*(NO) values in transgenic lines were significantly different from those in the wild type (Fig. 6D). These findings suggest that overexpression of *EjFAD2* enhances the stability of both the photosynthetic system and the plasma membrane under freezing stress.

The OJIP fluorescence kinetics were measured to evaluate differences in photosystem activity, and thereby clarifying the primary photochemical reaction of PSII and the electron transfer status within the photosynthetic machinery (Fig. 6E). The OJIP curves of transgenic lines were generally similar to those of the WT, but the values of Performance Index (*PI*abs) and photosynthetic driving force (*DF*abs) in transgenic plants were significantly higher than those in WT; these findings indicate that heterologous expression of *EjFAD2* enhances photosystem activity in *Arabidopsis*. Moreover, under freezing conditions, the shape of the OJIP curve in WT changed, with a notable increase in the ‘J’ step, suggesting that electron transport from QA to QB was impaired. Fluorescence parameter analysis further revealed that the values of *RE0*/CSm (quantum yield of electron transport flux per excited cross-section until PSI acceptors), *ET0*/CSm (electron transport flux per excited cross-section), *TR0*/CSm (trapped energy flux per excited cross-section), and *ABS*/CSm (absorption flux or effective antenna size per excited cross-section) were higher in transgenic lines than in WT. These results were consistent with data previously reported in loquat [28], supporting the conclusion that *EjFAD2* overexpression enhances photosystem performance in *Arabidopsis* under freezing stress.

In summary, the heterologous expression of *EjFAD2* helps maintain photosystem activity in *Arabidopsis* under freezing stress.

### EjMYBS3 interacts with EjFAD2 and EjMYBS3 contributes to enhanced freezing stress tolerance in *Arabidopsis*.

**Figure 7:**
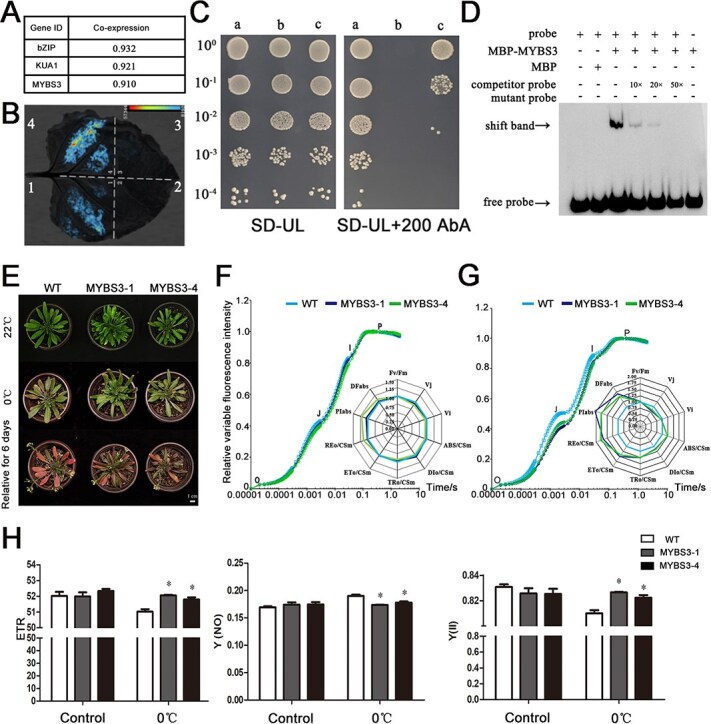
EjMYBS3 interacts with the EjFAD2 promoter and enhances freezing stress tolerance in *Arabidopsis*. (A) PCC values of genes co-expressed with *EjFAD2*. (B) DLR assays to verify EjMYBS3-mediated activation of the EjFAD2 promoter. 1, pGreenII-0800-LUC-FAD2 pro + pGreenII-62 SK; 2, pGreenII-0800-LUC + pGreenII-62 SK; 3, pGreenII-0800-LUC + pGreenII-62 SK-EjMYBS3; 4, pGreenII-0800-LUC-FAD2 pro + pGreenII-62 SK-EjMYBS3. (C) Y1H assay validating EjMYBS3 binding to the *EjFAD2* promoter. a, Positive, p53-AbAi x pGADT7-Rec53; b, pFAD2 pro-AbAi x pGADT7; c, pFAD2 pro-AbAi x pGADT7-EjMYBS3. (D) EMSA demonstrates that EjMYBS3 could bind to the EjFAD2 promoter. ‘−’ and ‘+’ indicate the lack and presence of the mentioned protein or probe, respectively. (E) Characterization of *EjMYBS3*-overexpressing transgenic lines under freezing stress conditions. (F) OJIP curves and fluorescence radar images of wild-type and *EjMYBS3-*overexpressing transgenic lines at 25°C. (G) OJIP curves and fluorescence radar images of wild-type and *EjMYBS3-*overexpressing transgenic lines at 0°C. (H) Modulated fluorescence parameters of *EjMYBS3-*overexpressing transgenic lines under freezing stress. The SD of three separate biological replicates is shown by error bars. Data underwent Student’s *t* test: ^*^ indicates *P* < 0.05.

To explore the regulatory factors of *EjFAD2*, we analyzed the Pearson correlation coefficient (PCC) values of genes co-expressed with *EjFAD2* and tabulated those with PCC values greater than 0.9 (Fig. 7A). Dual-luciferase reporter (DLR), yeast one-hybrid (Y1H) assay, and electrophoretic mobility shift assay (EMSA) confirmed that EjMYBS3 binds to the EjFAD2 promoter (Fig. 7B–D). To validate the functional role of EjMYBS3, overexpression lines were subjected to 0°C treatment. As shown in Fig. 7D, wild-type plants displayed more severe stress phenotypes both during cold exposure and after return to ambient temperature. Analysis of OJIP fluorescence kinetics and chlorophyll fluorescence parameters showed results consistent with those observed for EjFAD2, indicating that heterologous expression of *EjFAD2* enhances photosystem activity in *Arabidopsis* under freezing stress conditions (Fig. 7E–H) (Fig. S4B). Collectively, these findings demonstrate that EjMYBS3 interacts with EjFAD2, and EjMYBS3 enhances freezing stress tolerance in *Arabidopsis*.

### Transient overexpression of *EjFAD2* and *EjMYBS3* enhances freezing tolerance in loquat.

**Figure 8:**
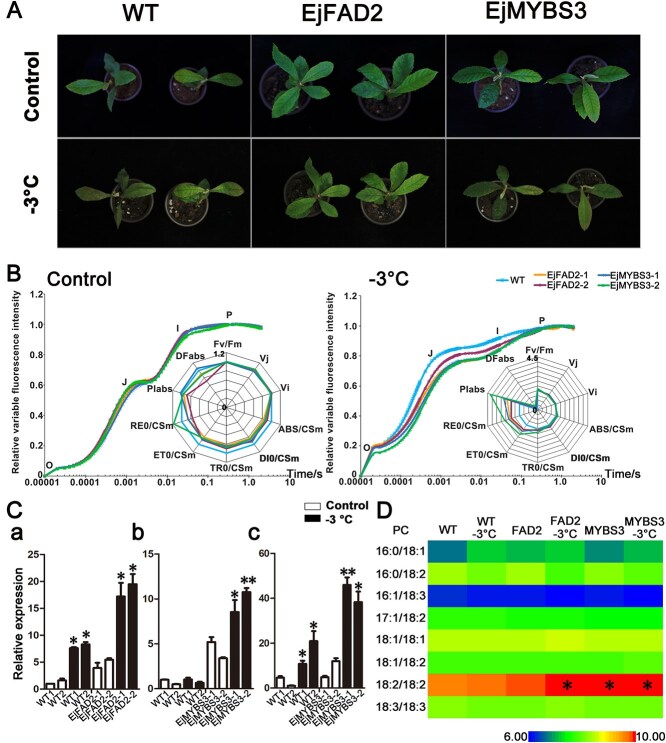
Overexpression of *EjFAD2* and *EjMYBS3* enhances freezing tolerance in loquat. (A) Characteristics of overexpression of EjFAD2 and overexpression of EjMYBS3 in loquat under −3°C. (B) OJIP curves and fluorescence radar images of wild-type and overexpressed transgenic lines under different temperatures. (C) Relative expression levels of *EjFAD2* and *EjMYBS3* in wild-type and overexpressed transgenic lines under different temperatures. a, Relative expression levels of *EjFAD2* in transiently overexpressing *EjFAD2* loquat by qPCR. b, Relative expression levels of *EjMYBS3* in transiently overexpressing *EjMYBS3* loquat by qPCR. c, Relative expression levels of *EjFAD2* in transiently overexpressing *EjMYBS3* loquat by qPCR. (D) Lipidomic analysis for wild-type and overexpressed transgenic lines under different temperatures; * indicates that FAD2 (−3°C), MYBS3, and MYBS3 (−3°C) are respectively compared with WT. The SD of three separate biological replicates is shown by error bars. Data underwent Student’s *t* test: ^*^ indicates *P* < 0.05.

To determine the functions of the EjFAD2 and EjMYBS3 in loquat, we performed transient transformation and exposed the plants to −3°C for 2 days. The results are shown in Fig. 8; overexpression of either *EjFAD2* or *EjMYBS3* enhanced the freezing stress tolerance of loquat. After freezing treatment, the expression level of *EjFAD2* was significantly higher in *EjFAD2*-overexpressing lines, while both *EjFAD2* and *EjMYBS3* expression levels were significantly elevated in *EjMYBS3*-overexpressing lines (Fig. 8C). Analysis of OJIP fluorescence kinetics and chlorophyll fluorescence parameters showed that overexpression of *EjFAD2* and *EjMYBS3* enhanced the photosystem activity of loquat under freezing stress (Fig. 8B). Lipidomic analysis was performed to further validate the effects of *EjFAD2* and *EjMYBS3* on loquat lipids. As shown in Fig. 8D, the levels of PC 18:2/18:2 in overexpression lines were all increased after freezing treatment and were significantly higher than those in WT controls.

In summary, the overexpression of *EjFAD2* and *EjMYBS3* elevated the content of PC 18:2/18:2, which contributed to the maintenance of photosystem activity in loquat under freezing stress. Consequently, the freezing tolerance of loquat was improved.

## Discussion

In this study, triploid loquat exhibited superior freezing tolerance compared to its parents. This finding aligns with previous reports demonstrating that polyploidy enhances stress tolerance in species such as *Brassica napus* [29], *Solidago canadensis* [30], *Trifoliate orange* [12], and *Fragaria moupinensis* [31]. Earlier research primarily focused on the seedless characteristic of triploids [32, 33]; however, triploids have also been shown to possess enhanced resistance and adaptability to environmental stresses [34], making the ploidy breeding a promising strategy for improving multiple agronomic traits [35]. Several factors may contribute to these observations, as polyploidization can induce extensive genomic and gene expression changes, including chromosomal rearrangements, chiasma formation, and gene deletions [36, 37]. Epigenetic modifications such as DNA methylation, gene silencing, and gene activation are also commonly observed in polyploids [38]. However, the regulatory mechanisms underlying freezing tolerance in triploids remain unclear, and further research is needed to elucidate the molecular basis of freezing resistance in triploids and to assess the potential of triploid breeding in improving stress tolerance.

As is well known, resistance and photosynthetic efficiency remain key factors limiting the yield and quality of the loquat. Freeze stress severely disrupts various photosynthetic processes, including light absorption and energy trapping, electron transport, and related biochemical reactions [39]. The chloroplast membrane plays a crucial role in maintaining chloroplast morphology and ensuring plant survival under freezing stress [40]. When plants are exposed to freezing conditions, chloroplasts are typically the first and most severely affected organelles. This includes observable changes such as thylakoid membrane fusion, disappearance of starch granules, and even chloroplast dissociation [41], as confirmed through transmission electron microscopy (TEM) observations in this study. At the same time, increased levels of fatty acid unsaturation in phospholipids help prevent freezing-induced membrane disruption [19]. Previous studies have demonstrated that unsaturated fatty acids play a significant role in plant tolerance to low-temperature stress. For instance, the *Arabidopsis fad2* mutant, which lacks endoplasmic reticulum-ω6 FAD, exhibits reduced polyunsaturated fatty acids in the extrachloroplast membrane and may even wither after prolonged exposure to cold stress [42]. Moreover, tobacco seedlings overexpressing the *Arabidopsis* chloroplast-ω3 *FAD7* gene showed enhanced freezing tolerance [42]. Therefore, we analyzed the lipid composition and degree of unsaturation, particularly focusing on unsaturated fatty acids, in loquat plants of different ploidies under freezing stress. The results indicated that triploids exhibited the most significant changes in lipid molecules. Notably, all three triploid lines showed the highest UI values for PC, especially for the PC 18:2/18:2 species. These findings suggest that PC 18:2/18:2 is a key contributor to the enhanced freezing tolerance observed in triploid loquat. Based on the observation that the unsaturation index (UI) of PC peaks among all lipids, we selected PC as the primary focus of this study. Concurrently, we noted that not only did the UI of PC increase significantly, but the UI values of other chloroplast membrane-associated lipids (including PE, PG, TAG, DGDG, SQDG, and MGDG) also rose markedly. The elevated unsaturation of TAG and PE contributes to enhanced plasma membrane stability, while the increased unsaturation indices of lipids such as PG, DGDG, SQDG, and MGDG improve the stability of chloroplast thylakoid membranes. PC was selected for further study due to its peak unsaturation level, with the highest value in triploids and the lowest in diploids, consistent with observed phenotypes. Meanwhile, the role of other lipids such as PG requires further investigation in loquat. These findings are also of considerable importance for future research on loquat’s response to freezing stress (Fig. S3).

FADs are key enzymes that regulate the levels of unsaturated fatty acid in plastid lipids. Unsaturated fatty acids play an essential role in maintaining membrane integrity under cold stress [43]. Our transcriptome analysis revealed that the *EjFAD2* gene displayed the most pronounced expression changes. This finding was further confirmed by real-time PCR, indicating that *EjFAD2* is an important gene involved in the cold stress response in triploid loquat. Lipid profiling of transgenic lines showed that the content of PC increased in all transgenic lines at 0°C, whereas it decreased in wild-type (WT) plants. Notably, PC levels were particularly elevated in *EjFAD2-*overexpressing lines, with a peak at PC 18:2/18:2, which is consistent with the lipid profiles observed in loquat. Heterologous expression of *EjFAD2* in *Arabidopsis* increased PC content, thereby enhancing the stability of leaf plasma membranes and photosynthetic membranes under freezing stress, as well as improving photosystem activity. Consequently, the freeze tolerance of the transgenic lines was significantly enhanced. Previous studies have demonstrated the involvement of FADs in plant cold stress responses; however, the transcriptional regulation of FADs remains to be fully elucidated. The identification of cold-induced MaMYB4 targeting multiple FADs, together with our observation that EjMYBS3 binds to the EjFAD2 promoter, suggests that MYB transcription factors are involved in the regulatory network of FADs [42]. Our results further demonstrate that overexpression of the *EjMYBS3* gene increases the expression level of *EjFAD2* in loquat, elevates the abundance of PC (18:2/18:2), and thereby enhances cold stress tolerance in loquat. These studies provide valuable target genes for molecular marker development and future molecular breeding in loquat. These results could also provide candidate genes for subsequent transformation and functional research. Furthermore, using transgenic materials as rootstocks for further investigation offers an effective tool for studying cold stress responses in loquat.

Compared to the parents, stronger heterosis in biomass, yield, resistance, and other agronomic traits is commonly observed in both diploid and polyploid organisms [44]. Previous studies have shown that heterosis is closely associated with differential gene expression rather than the emergence of novel genes [45, 46]. Heterosis can be better explained by additive and nonadditive gene expression patterns that lead to such phenotypic variations [47]. The expression level of *FAD2* was consistent with the freezing tolerance observed in the three ploidy levels of loquat, with the highest expression detected in triploid loquat compared to diploid and tetraploid types (Fig. 6). Sequence analysis of *FAD2* revealed identical gene sequences across ploidies, indicating that the observed nonadditive gene expression is not attributable to sequence variation. In recent years, several studies have advanced our understanding of fruit tree heterosis. For instance, in tetraploid *Poncirus trifoliata*, gene methylation has been found to correlate with enhanced cold tolerance [48]. Liu demonstrated that changes in gene expression patterns may result from DNA methylation and genetic variation, which may further contribute to heterosis in triploid loquat [31, 49, 50]. Further investigation into the regulatory mechanisms underlying heterosis will be essential to elucidate the molecular basis of frost resistance heterosis observed in this study.

**Figure 9:**
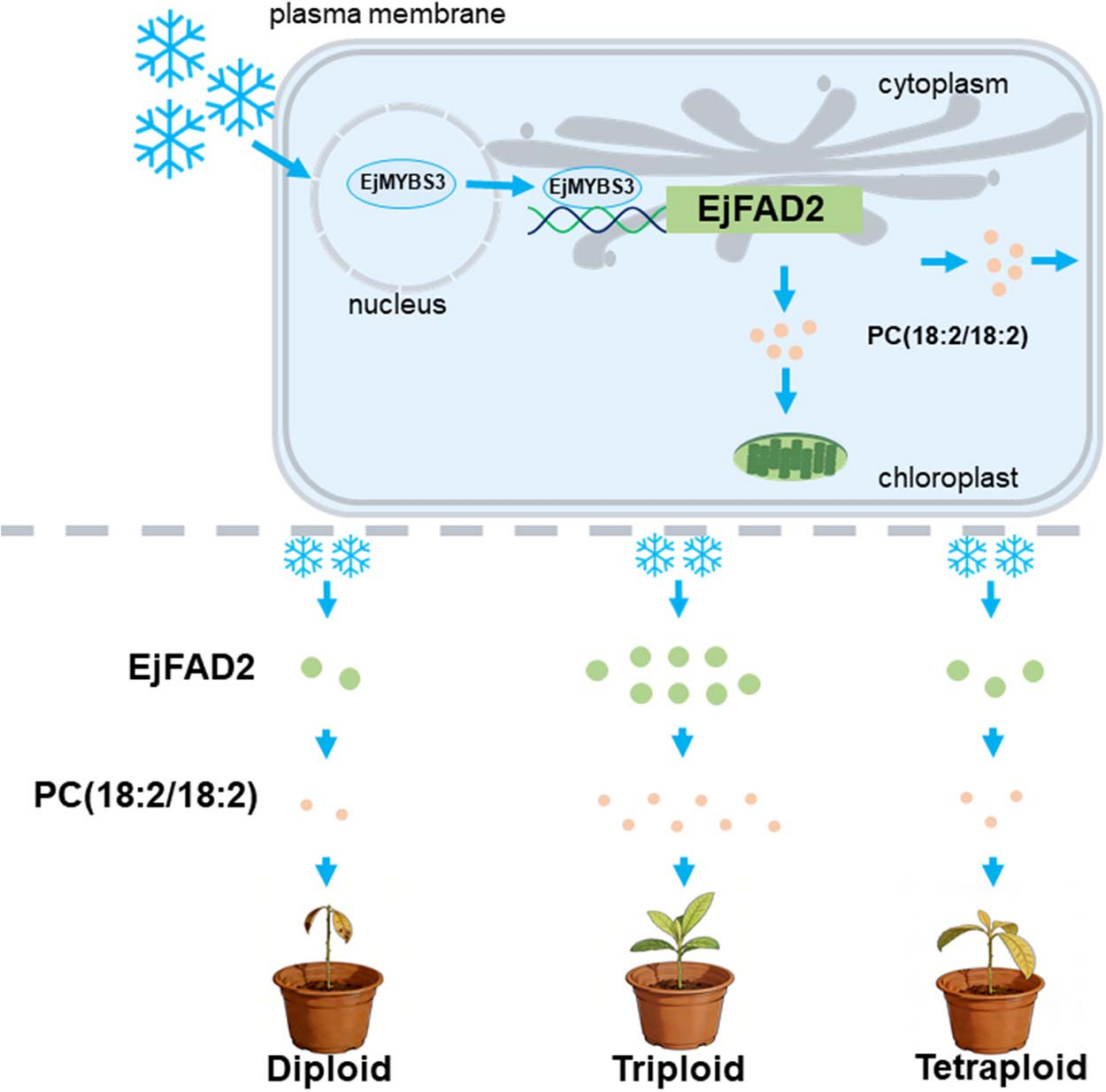
Molecular model of EjFAD2 regulation of freezing stress tolerance in loquat.

Collectively, these findings confirm that the molecular regulation module of EjFAD2 involved in loquat’s response to freezing stress. The transcription factor positively regulates the expression of EjFAD2, and the upregulation of EjFAD2 promotes the synthesis of PC (18:2/18:2), thereby maintaining photosynthetic stability and enhancing freezing tolerance in loquat (Fig. 9). These findings provide a theoretical foundation for understanding the mechanism underlying freezing stress tolerance in polyploid plants.

## Materials and methods

### Plant materials and growth conditions

All ploidy levels of loquats (*E. japonica* Lindl.) were grown in the field under standard agricultural practices and management at the loquat germplasm resource nursery (Southwest University, Chongqing, China). Specifically, the tetraploid loquat a spontaneous mutant of the cultivar Longquan 1 (B431), which served as the famale parent. The diploid loquat was obtained from the Guizhou wild loquat (GZ23), which served as the male parent. The triploid loquat was derived from the F1 generation of B431 × GZ23. All trees were grafted onto common cultivars used as rootstocks. The grafting was performed in February, and the treatments were conducted in December.

Specifically, the triploid lines B431 × GZ23-1, -2, and -3 respectively represent three independent lines of this strain.

Following ploidy analysis by flow cytometry after ploidy analysis, materials with different ploidy levels were labeled as follows: diploid, GZ23-1, -2, -3; triploid, B431 × GZ23-1, -2, -3; tetraploid, B431-1, -2, -3. Each ploidy group was subjected to temperature treatments of 0°C, −3°C, and −5°C for 72 h, respectively. With a control group maintained at 25°C. All other cultivation conditions remained consistent across treatments. The samples were then collected for further analysis, with three biological replicates conducted for each condition. *Arabidopsis thaliana* (ecotype Columbia) was used for genetic transformation. All homozygous transgenic lines were identified and selected for subsequent analysis. The wild-type plants and transgenic lines were grown under controlled conditions in a culture room (23°C, 16-h light, 70% humidity). The experiment was independently repeated three times.

### Isolation of total RNA and RT-PCR analysis

Total RNA was extracted from loquats samples subjected to different temperatures treatments using the cetyltrimethylammonium bromide acid (CTAB) phenol extraction method. Complementary DNA (cDNA) was synthesized from 20 μg of total RNA using TaKaRa’s cDNA synthesis kit, following the manufacturer’s instructions. The synthesized cDNAs were subsequently used as templates for real-time reverse transcription PCR (qRT-PCR) to analyze the expression levels of *EjFAD2* in loquat leaves. The genomic sequence information of *EjFAD2* was referenced from previous studies by Jiang and Jing [46, 47]. qPCR was carried out using Novostar-SYBR Supermix (Novoprotein, Shanghai, China). In addition, the relative expression levels of *EjFAD2* were calculated using the 2^−ΔΔCt^ method. All experiments were conducted with three biological replicates and three technical replicates.

### Plasmid construction and plant transformation

To construct a convenient plant expression vector, plasmid pFGC5941 was modified by inserting the CaMV 35S promoter–*EjFAD2*–NOS cassette, resulting in the renamed vector p*FGC5941*-*FAD2*. These plasmids were individually introduced into the *Agrobacterium* strain GV3101. Transformation of *Arabidopsis* was carried out using floral-dip method to generate the T_0_ transgenic lines, which were subsequently used to obtain the T_3_ generation for phenotypic observation and analysis.

### Transient expression assays

The *Agrobacterium* was grown at 28°C with shaking at 200 rpm until OD_600_ = 0.8–1.2 (OD_600_, optical density is at 600 nm). The bacterial cells were harvested by centrifugation at 5000 × *g* for 10 min. The pellet was resuspended in an infiltration buffer (10 mM MgCl_2_, 5 mM MES, and 200 μM acetosyringone). The suspension was then incubated at room temperature in the dark for 2 h. Uniformly grown loquat seedlings were inverted and immersed in the bacterial suspension. To facilitate entry, the leaves were gently punctured with a sterile needle prior to immersion. Vacuum infiltration was applied to promote the infiltration of the suspension into the leaf tissues. Following infection, the plants were kept in darkness for 48 h. Leaf samples were subsequently collected for expression analysis to identify lines successfully undergoing transient transformation.

### TEM staining report for loquat leaves specimen

The samples were sent to Chongqing Bonoheng Biotechnology CO., Ltd, to conduct TEM and analysis.

### Untargeted relative quantitative lipidomics

The samples were sent to Shanghai Biotree Biotech CO., Ltd, to conduct lipidomics analysis. The steps are as follows:

#### Metabolites extraction

Six groups were established: control and −3°C treatment for three types of loquats (diploid, triploid, tetraploid) with three biological replicates per group. A 30-mg sample was weighed into an EP tube for lipid metabolite extraction from loquat leaves. One hundred microliters of water and 400 μl of MTBE/MeOH (5:1, v/v) were added sequentially. The mixture was vortexed for 30 s, followed by homogenization at 35 Hz for 5 min and sonication for 5 min at 0°C; this process was repeated three times. After incubation at −40°C for 1 h, the samples were centrifuged at 3000 rpm for 15 min at 0°C. The pellet was discarded, and the supernatant was transferred to a new centrifuge tube and dried using a vacuum freeze concentrator. Subsequently, 200 μl of methanol/dichloromethane (1:1, v/v) was added, and the solution was immediately sonicated for 10 min at 4°C. Finally, after centrifugation at 13000 rpm for 20 min at 4°C, 75 μl of the supernatant was transferred to a fresh glass vial for LC/MS analysis.

#### LC–MS/MS analysis

Lipid analysis was performed using a UHPLC system (Agilent 1290, Agilent Technologies) equipped with a Kinetex C18 column coupled to mass spectrometry. The mobile phase consisted of two components: Phase A, which contained 10 mmol/l ammonium formate (HCOONH_4_) in ACN (acetonitrile)/H_2_O (6:4, v/v), and Phase B, which contained 10 mmol/l HCOONH_4_ in ACN/IPA (isopropanol) (1:9, v/v). The chromatographic separation was carried out following this gradient program:

1. 0-12  min: 60% A, 40% B, 300 μl/min.
2. 12-13.5 min, 100% B at a flow rate of 300 μl/min.
3. 13.5-18min, 60% A and 40% B at a flow rate of 300 μl/min.
4. 18-19 min, 60% A and 40% B at a flow rate of 300 μl/min.

The column temperature was maintained at 55°C, and the autosampler temperature was set to 4°C.

#### Data preprocessing

Output data were converted to mzXML format using ProteoWizard. Following analysis with XCMS, lipid identification was performed through accurate mass search and spectral matching against the LipidBlast library.

The unsaturation index (UI) was calculated according to Su *et al.* [48], using the following formula: UI = [sum of (*N* × mol%)]/100, where *N* represents the number of double bonds in each lipid molecular species and mol% refers to the molar percentage of a complex lipid class.

Abbreviations of lipids are as follows: ACar, acylcarnitine; AcylGlcADG, acylglucuronosyldiacyl-glycerol; BMP, bismonoacylglycerophosphate; CE, cholesteryl ester; Cer-NDS, ceramide non-hydroxyfatty acid-dihydrosphingosine; Cer-NS, ceramide non-hydroxyfatty acid-sphingosine; CL, cardiolipin; DAG, diacylglycerol; DGDG, digalactosyldiacylglycerol; DGTS, diacylglyceryl trimethylhomoserine; GlcADG, glucuronosyldiacylglycerol; HexCer-NDS, hexosylceramide non-hydroxyfatty acid-dihydrosphingosine; HexCer-NS, hexosylceramide non-hydroxyfatty acid-sphingosine; LPC, lysophophatidylcholine; MAG, monoacylglycerol; MGDG, monogalactosyldiacylglycerol; PC, phosphatidylcholine; PE, phosphatidylethanolamine; PEtOH, phosphatidylethanol; PG, phosphatidylglycerol; PMeOH, phosphatidylmethanol; PS, phosphatidylserine; SQDG, sulfoquinovosyl diacylglycerol; TAG, triacylglycerol.

### Transcriptome sequencing

The loquat leaf samples were collected on 22 March 2020, and subsequently stored at −80°C at Southwest University. Total RNA was extracted from the samples using the Tiangen RNAprep Pure Plant Plus kit, with additional steps taken to remove polysaccharides and polyphenols. RNA purification was further performed using the RNeasy Micro Kit in combination with the RNase-Free DNase Set. RNA purity was assessed using a spectrophotometer, while RNA integrity was evaluated using the Agilent 2100 Bioanalyzer. The qualified RNA samples were then submitted to Nanjing Personal Gene Technology Co., Ltd, for transcriptome sequencing, which yielded the transcriptome data. DEGs were identified based on a significance threshold of FDR < 0.05 and |log_2_ fold change) | ≥ 1. The analysis of DEGs was guided by a previously published methodology. Gene Ontology (GO) analysis (http://www.geneontology.org/) was conducted to assess the biological processes associated with each gene in the samples. The filtered reads were aligned to the reference genome of loquat (genome: pipachr.fa; Genebuild by GigaDB; Bases: 760132407).

Abbreviations are as follows: Cho, choline; P-Cho, phosphorylcholine; CDP-Cho, cytidine diphosphate choline; G3P, 3-phosphoglycerate; LPA, lysophosphatidic acid; PA, phosphatidic acid; DAG, diacyl glycerol; TAG, triacylglycerols; FA, fatty acids.

### Difference analysis of photosynthetic

#### Determination of photosynthetic parameters and chlorophyll fluorescence parameters

The first unfolded leaf to the fourth mature leaf (from top to bottom) was selected to study the photosynthetic and chlorophyll fluorescence parameters. After the temperature treatment of the materials, the photosynthetic parameters were measured by LCpro+ (ADC Bioscientific, UK). The red and blue light sources configured by the instrument were used for the measurement, and the PPFD was 250 μmol m^−2^ s^−1^. Chlorophyll fluorometer modulated by Junior-PAM (WALZ, Germany) was used to measure the fluorescence parameters. Three points were randomly selected from each leaf after dark adaptation for 20 min; *F*_0_ (minimum fluorescence), *Fm* (maximum fluorescence), *Y*(II) (Actual quantum efficiency), *Fv*/*Fm* (maximal quantum yield), *ETR* (electron transfer efficiency), qP (photochemical quenching coefficient), qN (Non photochemical quenching coefficient), *Y*(NO) (nonphotochemical quenching coefficient), *Y*(NPQ) (Non photochemical Quenching) were respectively measured.

#### Determination of light response curve

Three plants with the same growth potential were selected for each material, and the light response curve was measured by LCpro+ (ADC Bioscientific, UK). The light source is the red and blue light configured by the instrument, the measurement temperature is about 25°C, the carbon dioxide concentration is the atmospheric environment concentration, and the PPFD is 2000, 1800, 600, 1400, 1200, 1000, 500, 250, 200, 150, 100, 80, 40, 20, and 0, respectively.

#### OJIP curve

The OJIP curve of the same leaf was measured by using a multifunctional plant efficiency meter (M-PEA, Hansatech, UK).The operation steps were as follows: First, the leaves were clamped with dark-adapted leaf clips in the dark for 20 min. Then, the measuring light was turned on, and the fluorescence intensity was recorded at each time point from 10 μs for a total of 2 s. The moments corresponding to points O, J, I, and P on the OJIP curve are 0.02, 2, 30, and 400–1000 ms, respectively. Plot OJIP curves by using the mean fluorescence intensity of each material replicate and perform JIP-test analysis on OJIP curves.

Abbreviations are as follows: *RE0/*CSm (quantum yield of electron transport flux per excited cross-section until PSI acceptors)*, ET0/*CSm (electron transport flux per excited cross-section), *TR0/*CSm (trapped energy flux per excited cross-section), and *ABS/*CSm (absorption flux or effective antenna size per excited cross-section); V*i* or V*j*, the relative variable fluorescence at the *i* or *j* step; DIo, dissipation flux.

### Yeast one-hybrid assay

The EjFAD2 promoter fragments was inserted into the pAbAi vector; the full-length CDS of EjMYBS3 was cloned into the pGADT7. Both constructs were co-transformed into Y1HGold yeast strain and cultured on SD/-Leu/-Ura selection medium. Protein–DNA interactions between the EjFAD2 promoter and EjMYBS3 were assessed using Aureobasidin A (AbA) resistance assays.

### Determination of lipid peroxidation

The material was ground into a homogenate in phosphate-buffered saline (50 mM, pH 7.8) at 0°C–4°C, 10 000 g/15 min. Then determination of thiobarbituric acid reactive substances (TBARS) content as an indicator of leaf lipid peroxidation. Measure the absorbance at 450, 532, and 600 nm, and then calculate.


$$ C\ \left(\mathrm{\mu} \mathrm{mol}/\mathrm{l}\right)=6.45\ \left(\mathrm{A}532-\mathrm{A}600\right)-0.56\mathrm{A}450. $$

### Electrophoretic mobility shift assay

The *p*Mal-c5X vector was used to clone EjMYBS3 CDS, and the vector contains an MBP tag. We transformed *Escherichia coli Rosetta* cells and induced them to produce purified proteins. PlantCARE software was used to analyze the *cis*-acting elements within the EjMYBS3 promoter region to identify potential binding sites. Unlabeled competitive, mutated, and biotin-labeled probes were employed to evaluate the interaction between proteins and DNA (Table S2). The LightShift Chemiluminescent EMSA kit (Thermo) was used for EMSAs per the manufacturer’s protocol.

## Supplementary Material

Web_Material_uhag096

## Data Availability

All relevant data can be found within the article and its supporting materials. Data link (Lipidome of loquat): https://www.scidb.cn/s/ZZzQ7j; Data link (Transcriptome of loquat): https://www.scidb.cn/anonymous/Wlp6UTdq.
