# Supplementary material for: Triploid loquat maintains photosynthetic stability under freezing stress through excessive accumulation of unsaturated lipids
Source: Hortic Res. 2026 Mar 16;13(7):uhag096. doi: 10.1093/hr/uhag096 (PMC13271775; doi:10.1093/hr/uhag096)
Supplement: Web_Material_uhag096 [file web_material_uhag096.docx]

**Supplementary**

**Table S1. Low Temperature LT50 of different ploidy lines.**

| Materials | REC assessment | | Fv/Fm assessment | |
| --- | --- | --- | --- | --- |
|  | Fitting degree R^2^ | Semilethal temperature LT50/℃ | Fitting degree  R^2^ | Semilethal temperature  LT50/℃ |
| GZ23 | 0.9729 | -6.545 | 0.9816 | -9.972 |
| B431 × GZ23 | 0.9874 | -9.419 | 0.9816 | -13.35 |
| B431 | 0.9724 | -7.195 | 0.9903 | -9.459 |

**Table S2. Primers used in EMSA.**

| Name | Primer | Labeling |
| --- | --- | --- |
| MYBS3 F | aatttgttatctcatatata | 5'-Biotin labeling |
| MYBS3 R | tatatatgagataacaaatt |  |
| MYBS3 com F | aatttgttatctcatatata |  |
| MYBS3 mutant F | aatttgggggggcatatata | 5'-Biotin labeling |
| MYBS3 mutant R | tatatatgcccccccaaatt |  |


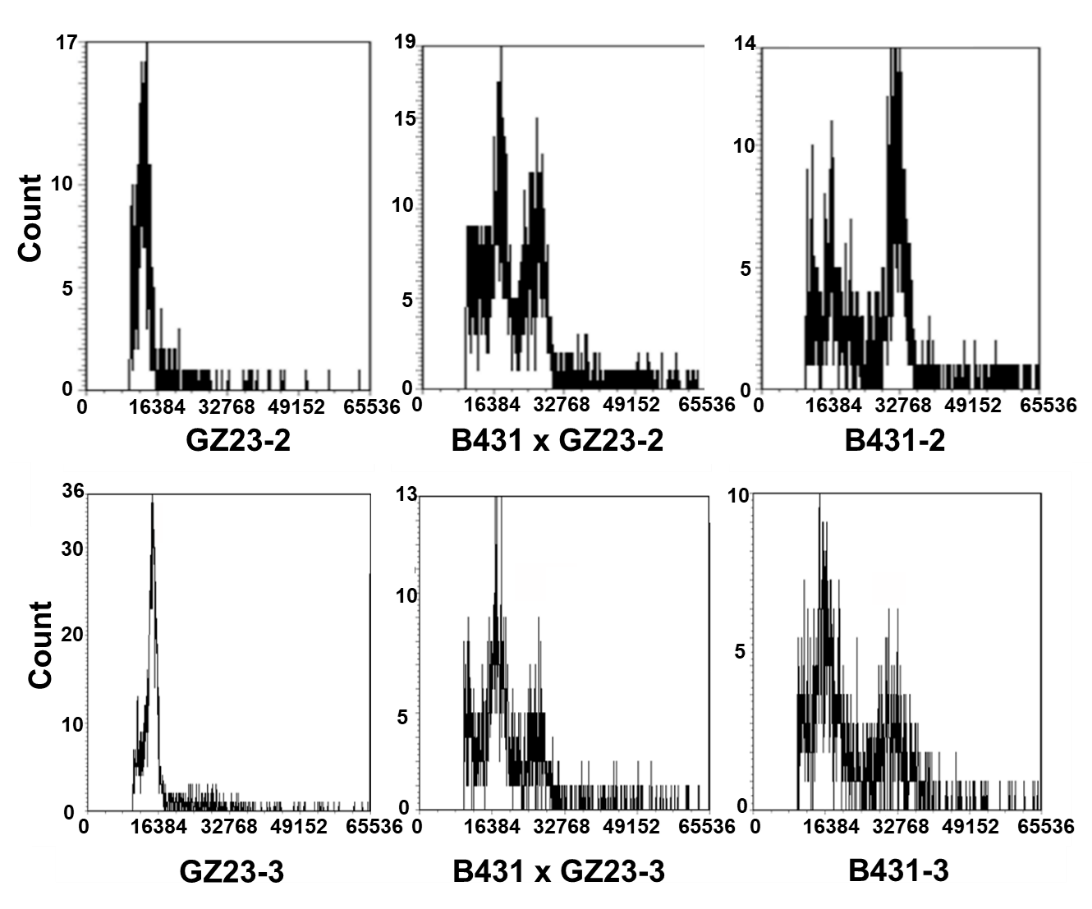


**Figure S1.** **Flow cytometric histograms of DAPI-stained leaf nuclei of different ploidy loquat.**

Diploid, GZ23-2, GZ23-3; Triploid, B431 × GZ23-2, B431 × GZ23-3; Tetraploid, B431-2, B431-3.

**
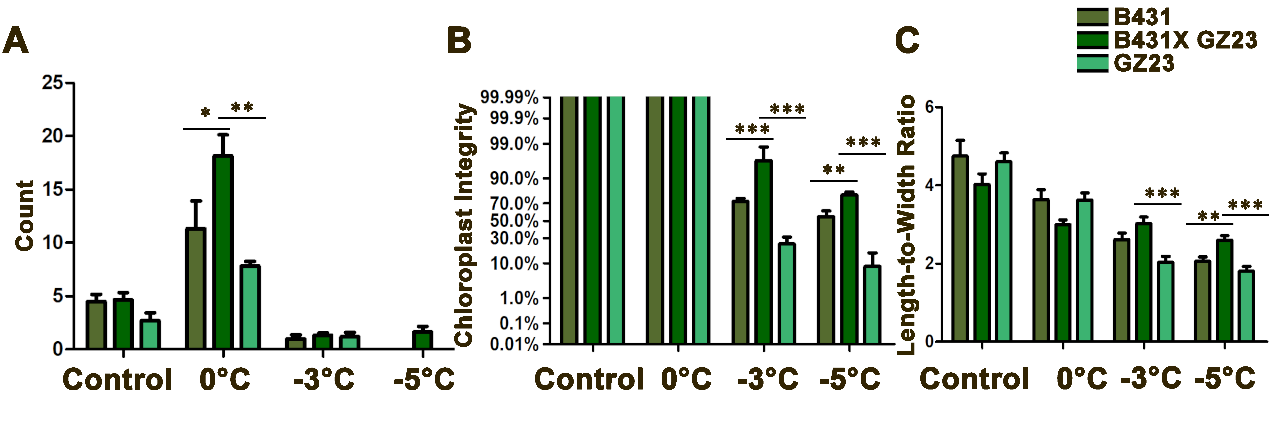
**

**Figure S2. Statistical analysis of cellular ultrastructure in loquat of different ploidy levels under freezing stress. (A), Starch granule number.** The number of starch granules in individual intact cells was quantified. **(B), Chloroplast Integrity.** **(C), Chloroplast Length-to-Width Ratio.** The number of intact chloroplasts and their corresponding length and width within individual intact cells were quantified.

The SD of three separate biological replicates is shown by error bars. Data underwent Student’s test: **, *** indicate *p* <0.01, *p* < 0.001.


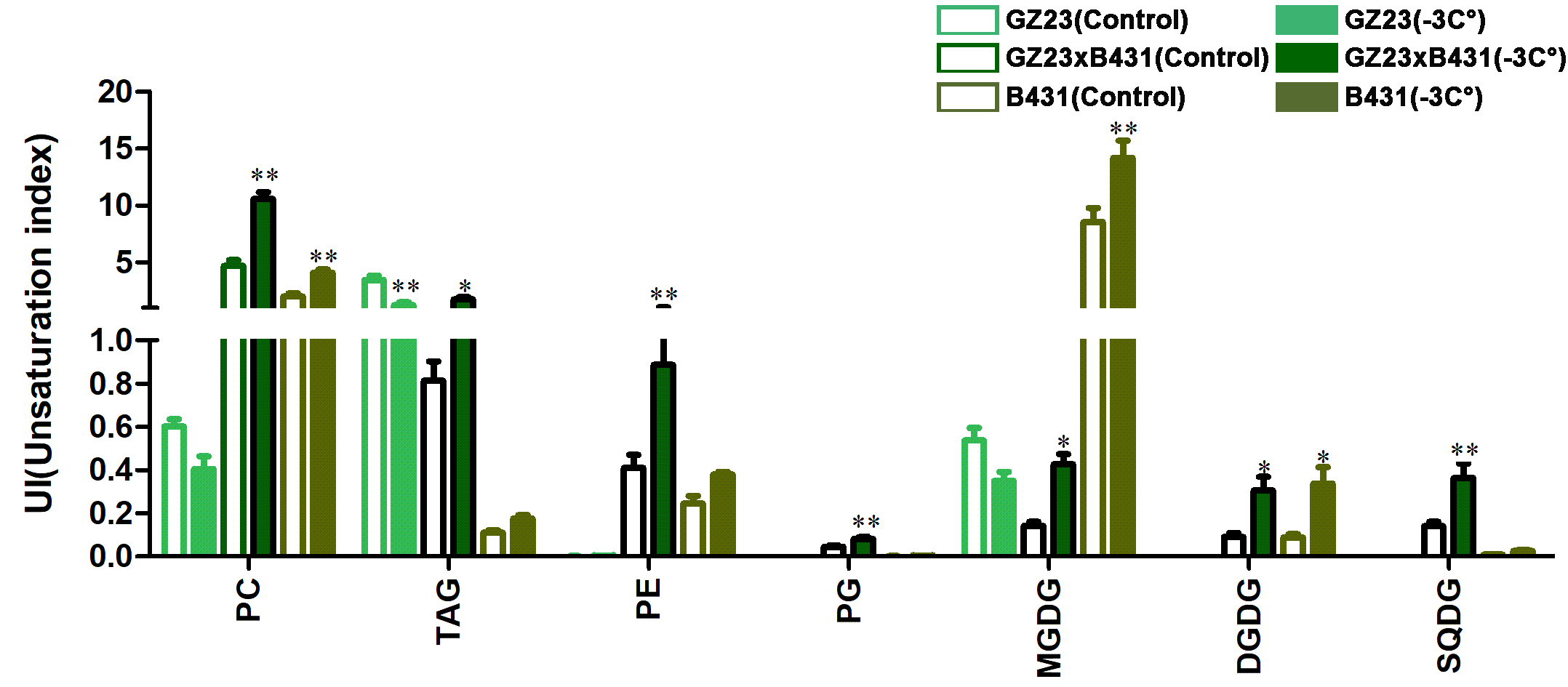


**Figure S3. The unsaturation index (UI) of different lipids in each ploidy loquat under freezing stress.**

The SD of three separate biological replicates is shown by error bars. Data underwent Student’s test: *, ** indicate *p* <0.05, *p* < 0.01.

**

**

**Figure S4.** **The identification of overexpressing** ***EjFAD2* and *EjMYBS3* transgenic *Arabidopsis*.** **(A), Expression levels of *EjFAD2* in overexpressing *EjFAD2* transgenic lines leaves. (B), Expression levels of *EjMYBS3* in overexpressing *EjMYBS3* transgenic lines leaves.**

The SD of three separate biological replicates is shown by error bars. Data underwent Student’s test: **, *** indicate *p* <0.01, *p* < 0.001.
